# A man with dilated superficial abdominal veins: A clinical presentation of non-Hodgkin lymphoma

**Published:** 2016

**Authors:** Khalid Hamid Changal, Sheikh Shoaib Altaf, Adnan Raina

**Affiliations:** 1 Internal Medicine, Sher-i-Kashmir Institute of Medical Sciences, Srinagar, India.; 2 Mercy Catholic Medical Center, Philadelphia, USA.

**Keywords:** Abdominal vein, None-hodgkin lymphoma, Vena cava

## Abstract

**Background:**

The clinical presentation of non-Hodgkin lymphoma (NHL) varies tremendously depending upon the type of lymphoma and the areas of involvement. NHL can rarely present as an abdominal mass compressing the inferior vena cava. The clinical presentation due to obstruction of inferior vena cava has often been called the inferior vena cava syndrome (IVCS). It can present acutely or chronically.

**Case Presentation:**

A 35-year-old male presented with 3 months history of fever, anorexia, weight loss and abdominal discomfort. His abdominal examination showed dilated superficial veins with blood flowing rostrally. CECT abdomen revealed multiple enlarged some necrotic, retroperitoneal lymph nodes. The inferior vena cava was noted to be compressed by the lymph nodes. The lymph node biopsy revealed non- Hodgkin lymphoma, precursor B cell.

**Conclusion:**

An abdominal mass compressing the inferior vena cava is a rare but possible cause for appearance of dilated superficial abdominal veins and should be looked for.

NHL varies tremendously depending upon the type of lymphoma and the areas of involvement. Inferior vena cava (IVC) obstruction can result from thrombosis secondary to hypercoagulable disorders, extrinsic compression by tumors, infective phlebitis, inflammation, trauma, surgery, or in many number of cases, idiopathic (1-4). When NHL presents as an abdominal mass compressing the inferior vena cava, the focus is to acutely prevent deep venous thrombosis or pulmonary embolism. Following this, chemotherapy to control lymphoma is important (4).

## Case presentation

**Figure 1:**
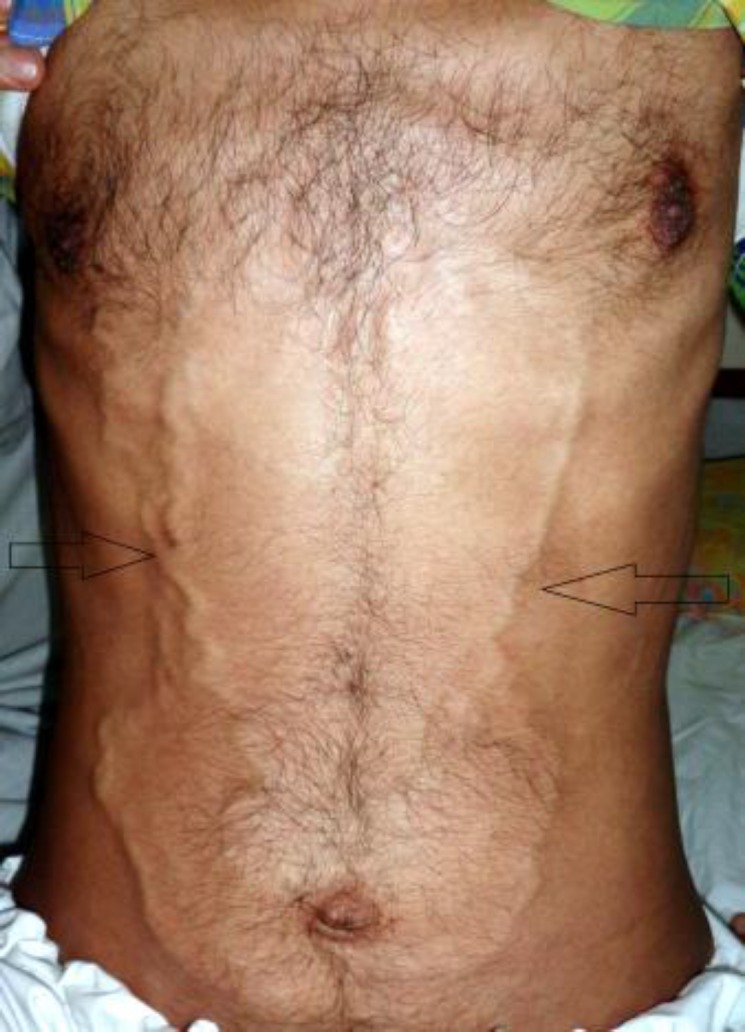
An image showing dilated and tortuous superficial abdominal veins

**Figure 2:**
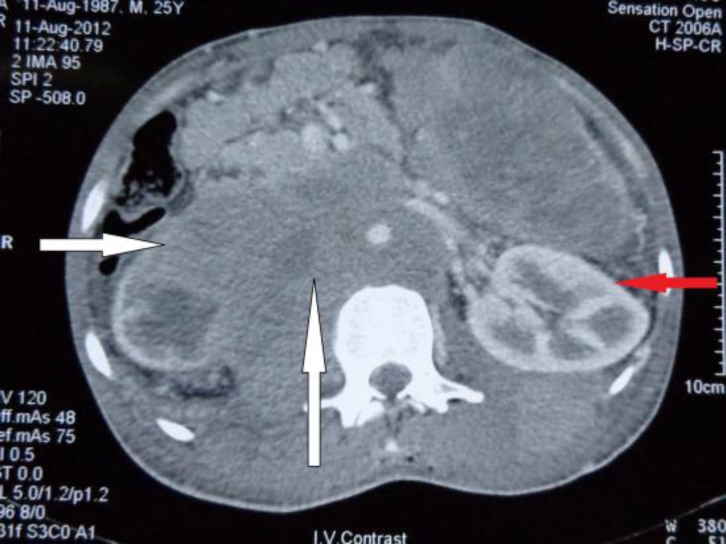
A CECT of the abdomen showing lymph nodal mass compressing the inferior vena cava (vertical white arrow). The right kidney has also been compressed (horizontal white arrow), the left kidney is normal (horizontal red arrow

A 35-year-old male presented with 3 months history of fever, anorexia, weight loss and abdominal discomfort in the month of August 2012 to the Department of Medicine at SKIMS, Soura. He was emaciated. His abdominal examination showed dilated superficial veins with blood flowing rostrally (figure1). Hepatosplenomegaly was noted. An ultrasonogram of the abdomen showed multiple enlarged retroperitoneal lymph nodes. Investigations showed lymphocytosis and anemia. The cause of the dilated abdominal veins was still not evident. A contrast enhanced computed tomography (CECT) of the abdomen was done. The CECT revealed multiple enlarged some necrotic, retroperitoneal lymph nodes (figure 2).

**Figure 3:**
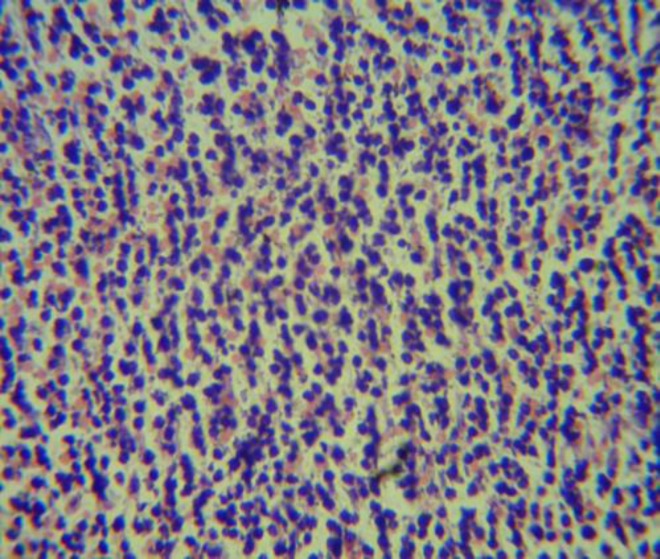
A photomicrograph of the lymph node showing NHL (stain used: hematoxylin and eosin

The inferior vena cava was noted to be compressed by the lymph nodes causing multiple collateral blood vessels to open up in compensation and thus manifesting on the skin of the abdomen as well. An image guided biopsy of one of the lymph nodes was done and the patient was given enoxaparin to prevent deep venous thrombosis of lower limbs and abdominal veins. The lymph node biopsy revealed non- Hodgkin lymphoma, precursor B cell (figure 3). Patient was started on chemotherapy (cyclophosphamide, doxorubicin, vincristine, and prednisone) but succumbed to sepsis and multiorgan failure after 3 months. An abdominal mass compressing the inferior vena cava is a rare but possible cause for appearance of dilated superficial abdominal veins and should be looked for. 

## Discussion

Non-Hodgkin lymphoma (NHL) is a collective term for a heterogeneous group of lymphoproliferative malignancies with differing patterns of behavior and responses to treatment. (1) The clinical presentation of NHL varies tremendously depending upon the type of lymphoma and the areas of involvement. Some NHLs behave indolently with lymphadenopathy waxing and waning over years. Others are highly aggressive, resulting in death within weeks if left untreated. Approximately 50 percent of patients will develop extranodal disease (secondary extranodal disease) while between 10 and 35 percent of patients will have primary extranodal lymphoma at initial diagnosis. (2) Involvement of retroperitoneal, mesenteric, and pelvic nodes is common in most histologic subtypes of NHL. Unless massive or leading to obstruction, nodal enlargement in these sites usually does not produce symptoms. (3, 4)

Inferior vena cava (IVC) obstruction can result from thrombosis secondary to hypercoagulable disorders, extrinsic compression by tumors, infective phlebitis, inflammation, trauma, surgery, or in many number of cases, idiopathic. (5, 6) The clinical presentation due to obstruction of inferior vena cava has often been called the inferior vena cava syndrome (IVCS). It can present acutely or chronically. IVCS presents as edema of lower limbs, deep venous thrombosis of lower limbs and/or as dilated superficial and visceral collateral veins to allow venous blood from the legs to return to the right atrium (7). It is important to clinically assess the flow of blood in the dilated veins. In patients with dilated abdominal wall veins due to cirrhosis, the direction of blood flow is away from the umbilicus (radiating like a star from the umbilicus), whereas in vena caval obstruction, the direction of blood flow is either completely above downward (superior vena cava obstruction) or completely below upward (inferior vena cava obstruction) (8).

Precursor B-cell acute lymphoblastic leukemia/ lymphoma is a neoplasm of lymphoblasts committed to the B-cell lineage, typically composed of small to medium-sized blast cells with scant cytoplasm, moderately condensed to dispersed chromatin and indistinct nucleoli, involving bone marrow and blood (acute lymphoblastic leukemia), and occasionally presenting with primary involvement of nodal or extranodal sites (lymphoblastic lymphoma). When presenting as an abdominal mass compressing the inferior vena cava, the focus is to acutely prevent deep venous thrombosis or pulmonary embolism. 

Following this chemotherapy to control lymphoma is important. Both these goals of interest are usually started simultaneously in the form of heparin and chemotherapy. Aggressive supportive therapy is required to prevent the complications of chemotherapy. In our case, although we were able to prevent deep venous thrombosis, the patient succumbed to sepsis due to chemotherapy-related complications. 

## Citation:

Changal KH, Altaf SS, Raina A. A man with dilated superficial abdominal veins. A clinical presentation of non-Hodgkin lymphoma. Caspian J Intern Med 2016; 7(3): 222-224.
